# Effects of rebamipide for chronic atrophic gastritis: A protocol for systematic review and meta-analysis

**DOI:** 10.1097/MD.0000000000020620

**Published:** 2020-06-19

**Authors:** Daorui Hou, Maoyi Yang, Zhipeng Hu, Liangjun Yang

**Affiliations:** a Department of Traditional Chinese Medicine Oncology, The First People's Hospital of Xiangtan City, Xiangtan, Hunan Province; b Hospital of Chengdu University of Traditional Chinese Medicine, Chengdu, Sichuan Province; c Department of Gastroenterology, Tongde Hospital of Zhejiang Province, Hangzhou, Zhejiang Province, China.

**Keywords:** chronic atrophic gastritis, protocol, rebamipide, systematic review and meta-analysis

## Abstract

**Background:**

Chronic atrophic gastritis (CAG) is defined as an important precancerous disease with high risk of gastric cancer. Rebamipide is a mucosal protective agent widely used in the treatment of chronic gastritis. The aim of this systematic review is to assess the efficacy and safety of rebamipide for the treatment of patients with CAG.

**Methods and analysis:**

We will perform a comprehensive retrieval in the following electronic databases: PubMed, Embase, MEDLINE, Cochrane Library Central Register of Controlled Trials, China National Knowledge Infrastructure (CNKI) database, Wanfang Data Knowledge Service Platform, Chinese Scientific Journals Database (VIP), Chinese Biomedical Literature Service System (SinoMed) and other sources. Two trained researchers will select the qualified studies for data extraction and assess the quality and risk of bias, independently. Then the meta-analyses will be conducted by using the RevMan 5.2 and stata 14.0. The heterogeneity of data will be investigated by Cochrane X^2^ and *I*^*2*^ tests. Sensitivity analysis will be conducted to evaluate the stability of the results. Funnel plot analysis and Egger test will be used to assess the publication bias. Finally, the quality of evidence will be assessed by the GRADE system.

**Results:**

The results of our research will be published in a peer-reviewed journal.

**Conclusion:**

The conclusion of our systematic review will provide evidence to judge whether rebamipide is an effective intervention for patient with CAG.

**OSF registration number:**

10.17605/OSF.IO/BKC3E.

## 1 Introduction

Chronic atrophic gastritis (CAG), a chronic inflammation of the gastric mucosa, is associated with the loss of gastric gland cells and replaced by intestinal epithelium and fibrous tissue. It is usually defined as an important precancerous disease^[1]^ and is associated with an increased risk for gastric cancer,^[2]^ one of the most common and aggressive tumors of the digestive tract around the world. It is generally accepted that the model for gastric cancer development begins with gastritis, proceeds into CAG, then develops into intestinal metaplasia, dysplasia, and finally to cancer.^[3]^ Moreover, according to a multi-center national study in China, CAG accounted for 25.8% of patients with gastritis.^[4]^ Therefore, effective intervention of CAG is an important method to prevent the occurrence of gastric cancer.

*Helicobacter pylori* infection is a leading etiological factor for CAG, and is defined as a class I carcinogen through trigging a progression from CAG to gastric carcinogenesis.^[5,6]^ Although the triple and quadruple therapies are wildly used as the first-line treatment of *H. pylori* infection. The management of *H. pylori* has now become a challenge owing to the emergence of antibiotic-resistant *H. pylori* strains.^[7]^ Additionally, acid-suppressing drugs, mucosal protective agents, and prokinetic agents are commonly used to alleviate the clinical symptoms and reduce the gastric mucosal damage in the treatment of CAG.^[8]^ But the medications still cannot meet clinical needs with respect to reduce the histopathological injuries. Other drugs like COX inhibitors, nonsteroidal anti-inflammatory drugs, and antioxidant vitamins are also used to treat CAG with intestinal metaplasia or dysplasia.^[9]^ However, the efficacy and safety of these therapies are still not satisfactory.^[9–11]^

Rebamipide is an amino acid derivative used for the treatment of gastritis and gastric ulcer. Studies have demonstrated that rebamipide enhances gastric mucosal protection by increasing endogenous prostaglandin production and inhibiting free radical production.^[12,13]^ Additionally, it has anti-inflammatory effects by inhibiting cytokines and impeding neutrophil activation.^[14,15]^ Furthermore, a clinical study has demonstrated that rebamipide improves the clinical symptoms of patients with chronic gastritis and reduces gastric mucosal lesions.^[16]^ A meta-analysis which focused on the efficacy of rebamipide for chronic gastritis indicated that rebamipide combined with conventional treatments could improve patients’ symptom compared to the treatment with conventional medicine.^[17]^ However, the pathological changes associated with chronic gastritis were not measured. Therefore, a comprehensive review is urgently needed to support the effectiveness and safety of rebamipide on patients with CAG. In this work, we will systematically evaluate the clinical efficacy of rebamipide for CAG using a meta-analysis method, thereby providing an objective and scientific basis for clinical practice.

## 2 Methods and analysis

This systematic review will be conducted followed the guideline of the Preferred Reporting Items for Systematic Review and Meta-Analysis Protocols (PRISMA-P) recommendations.^[18]^ This work has been registered at Open Science Framework (OSF, https://osf.io/), an open source project management that helps in the design of studies. The registration DOI of this study is 10.17605/OSF.IO/BKC3E.

### 2.1 Eligibility criteria

#### 2.1.1 Study design

Randomized controlled trials (RCTs) which used rebamipide or a combination of rebamipide and routine pharmacotherapy as treatment measures will be eligible. Non-randomized control studies and observational study will be excluded in the review. Language will be limited to English and Chinese.

#### 2.1.2 Type of participants

This study will include patients diagnosed with CAG by endoscopic assessment and mucosal biopsy. There will be no limitation about age, gender, region, and other factors. Individuals with other digestive diseases, such as peptic ulcer or gastric cancer will be excluded.

#### 2.1.3 Interventions/comparators

We will only include studies which interventions involved rebamipide alone or combined with other routine pharmacotherapy, as well as those with control groups which can verify the therapeutic effect of rebamipide. Studies where the control group is different from the therapy in the intervention group will be excluded. Trials in which the control group will include placebo control, no treatment, and conventional treatments, such as proton pump inhibitor, antibiotics, or prokinetics. The treatment durations should be at least 8 weeks.^[19,20]^

### 2.2 Outcomes

#### 2.2.1 Primary outcomes

The improvement of gastric histopathology is considered the primary outcome to evaluate therapeutic effects.

#### 2.2.2 Secondary outcomes

The secondary outcomes will include:

1. The improvement of clinical symptoms, such as distension, upper abdominal pain, acid reflux, belching, loss of appetite, etc.
2. The curative effect of endoscopic.
3. The clearance of *H. pylori*.
4. Adverse events.

### 2.3 Search strategy

#### 2.3.1 Electronics searches

To identify all relevant studies, a comprehensive electronic search of the following databases will be performed, including PubMed, Embase, MEDLINE, Cochrane Library Central Register of Controlled Trials, China National Knowledge Infrastructure (CNKI) database, Wanfang Data Knowledge Service Platform, Chinese Scientific Journals Database (VIP), Chinese Biomedical Literature Service System (SinoMed) from their inception to April 2020. The following subject terms and key words will be used in the search: (“atrophic gastritis” OR “gastritis, atrophic” OR “ chronic atrophic gastritis” OR “chronic gastritis” OR “gastric atrophy” OR “gastric mucosal atrophy” OR “CAG”) and (“rebamipide” OR “OPC-12759” OR” pramipide” OR” proamipide” OR” mucosta”) and (“randomized controlled trial” OR “RCT” OR “random” OR “randomly” OR “random allocation” OR “allocation” OR “randomized control trial” OR “controlled clinical trial” OR “clinical trial” OR “clinical study” OR “placebo”). The equivalent search words will be used in the Chinese databases. The search strategy for PubMed is shown in Table 1.

**Table 1:**
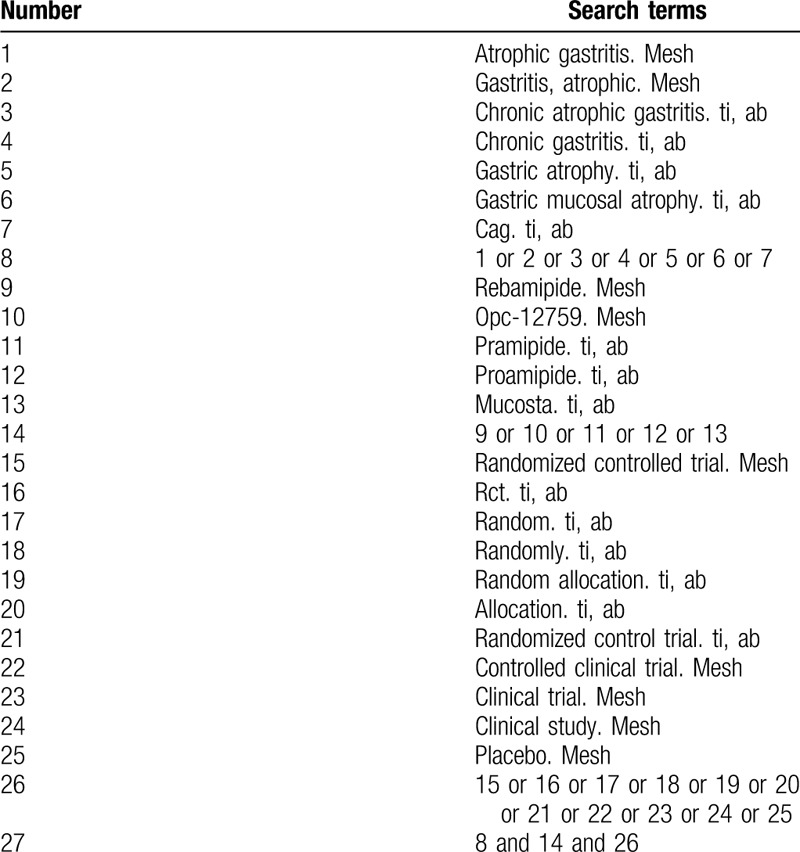
Search strategy used in PubMed.

#### 2.3.2 Searching other resources

Studies from other sources will also be obtained from the following sources:

1. Google scholar and Baidu scholar.
2. WHO International Clinical Trial Registry Platform
3. Chinese Clinical Trial Registry (ChiCTR).
4. ClinicalTrials.gov.

### 2.4 Data collection and analysis

#### 2.4.1 Selection of studies

**Figure 1:**
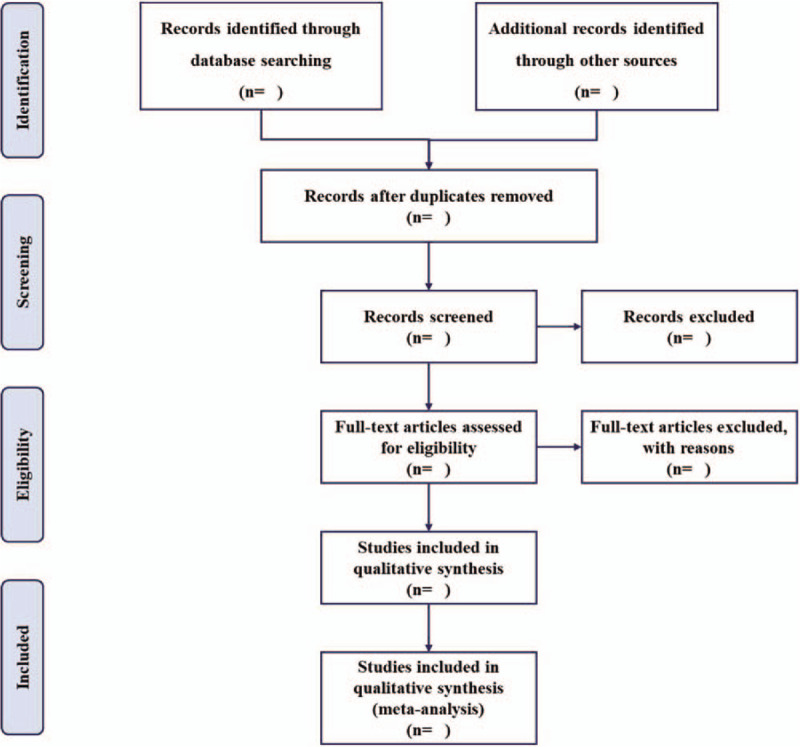
Flow chart of study selection.

The electronic citations extracted out from the above databases will be managed by EndNote X9.0 software. The titles and abstracts of all searched studies will be assessed independently by 2 methodological trained reviewers (DH and ZH) in accordance with the established selection criteria. The full-text papers will be reviewed if necessary. Any disagreements generated between the 2 reviewers will be arbitrated through consensus with the corresponding author. Excluded studies will be listed in a table with reasons. A PRISMA flow chart will be drawn to illustrate the study selection procedure (Fig. 1).

#### 2.4.2 Data extraction and management

The information extracted from all eligible studies by 2 independent reviewers (DH and ZH) will be checked. The data of those qualified articles will be export to Excel table, which includes the first authors of the article, publication year, pathological type of gastric, interventions in experimental group and control group, time of treatment, ample size in each group, age, gender, outcome indicators, and adverse events. When data are not available from the studies, the missing information will be obtained by contacting the corresponding authors.

#### 2.4.3 Assessment of risk of bias

The risk of bias for each study will be evaluated by 2 independent reviewers DH and ZH) using the Cochrane Collaboration's tool.^[21]^ In this tool, the risk of bias of a trial is assessed through 6 items, including selection bias, performance bias, detection bias, attrition bias, reporting bias and other sources of bias. The assessment will be classified into 3 levels: “Low risk”, “High risk” or “Unclear risk.” Any disagreements between the 2 reviewers will be resolved by discussion of all reviewers.

#### 2.4.4 Measures of treatment effect

The data will be analyzed by RevMan 5.2 (Cochrane, London, UK) and stata 14.0 software. For dichotomous variables, a risk ratio (RR) with 95% confidence interval (CI) will be used for analysis. For continuous variables, a mean difference (MD) or a standard mean difference (SMD) with 95% CIs will be used for analysis. MD will be used when the treatment outcome was measured by the same scale. SMD will be used when the treatment outcome was measured by different scales in different studies.

#### 2.4.5 Assessment of heterogeneity

To assess the statistical heterogeneity of evidence, the Cochrane *X*^*2*^ and *I*^*2*^ tests will be utilized.^[22]^ If *P* ≥ .05 and *I*^2^≤50%, it suggests that no statistical heterogeneity is observed between subgroups. Then the fixed effect model will be applied to estimate the effect amount. If *P* < .05 and *I*^*2*^ > 50%, it is considered that there is great heterogeneity between the studies, and the random effect model will be used. The results will be showed in tables and figures when the quantitative synthesis is not suitable.

#### 2.4.6 Sensitivity analysis

A sensitivity analysis will be performed to evaluate the robustness of the results. We will exclude each study included in the analysis. Then we will re-analyze and compile the data. The difference between the re-obtained effects and the original effects will be compared.

#### 2.4.7 Assessment of reporting biases

A funnel plot will be drawn to assess the publication bias when more than 10 studies are included in the meta-analysis. The potential reporting biases will be statistical appraised by the Egger test.^[23]^

#### 2.4.8 Grading the quality of evidence

The Grading of Recommendations Assessment, Development and Evaluation (GRADE) will be applied to evaluate the quality level of evidence.^[24]^ The assessments of evidence quality will be assorted into “high”, “moderate”, “low”, and “very low” quality.^[25]^

### 2.5 Patient and public involvement

Patient and public were not involved in this study.

### 2.6 Ethics and dissemination

Ethical approval will not be required for this systematic review because the data used are not linked to individual patient. The results of this review will be disseminated by being published in a peer-reviewed journal.

## 3 Discussion

CAG is an important precursor lesion of gastric cancer, a major health problem with high morbidity and mortality in China.^[26]^ Early intervention should be emphasized in the management of CAG. Rebamipide is a mucosal protective agent widely used in the treatment of gastritis. Several studies have demonstrated that the application of rebamipide during the eradication of *H. pylori* can improve the eradication rate,^[27]^ and is effective in improving symptoms in patients with functional dyspepsia.^[28]^ However, there is no systematic and comprehensive clinical evidence to summarize the clinical efficacy of rebamipide on CAG, especially for its effect on gastric mucosal pathology, which limits the clinical application of this drug. Thus, we decide to conduct this meta-analysis to evaluate the efficacy and safety of rebamipide for patients with CAG. We hope that this review will provide a better guide for clinicians when dealing with CAG.

### 3.1 Amendments

If amendments are needed, we will update our protocol to include any changes in the whole process of research.

## Author contributions

**Conceptualization:** Liangjun Yang.

**Data curation:** Daorui Hou, Zhipeng Hu, Maoyi Yang.

**Formal analysis:** Maoyi Yang.

**Funding acquisition:** Daorui Hou.

**Investigation:** Daorui Hou, Zhipeng Hu.

**Methodology:** Daorui Hou, Zhipeng Hu, Maoyi Yang.

**Project administration:** Liangjun Yang.

**Resources:** Daorui Hou, Zhipeng Hu.

**Software:** Daorui Hou, Zhipeng Hu.

**Supervision:** Liangjun Yang.

**Writing – original draft:** Liangjun Yang, Zhipeng Hu.

**Writing – review & editing:** Zhipeng Hu, Liangjun Yang.
